# Cell-specific prediction and application of drug-induced gene expression profiles

**Published:** 2018

**Authors:** Rachel Hodos, Ping Zhang, Hao-Chih Lee, Qiaonan Duan, Zichen Wang, Neil R. Clark, Avi Ma'ayan, Fei Wang, Brian Kidd, Jianying Hu, David Sontag, Joel Dudley

**Affiliations:** 1 Institute for Next Generation Healthcare, Icahn School of Medicine at Mount Sinai (ISMMS), New York, NY, 10065; 2 Department of Genetics and Genomic Sciences, ISMMS, New York, NY, 10029; 3 Courant Institute of Mathematical Sciences, New York University, New York, NY, 10012; 4 IBM T. J. Watson Research Center, Yorktown Heights, NY, 10598; 5 Dept. of Pharmacological Sciences, ISMMS, New York, NY, 10029; 6 BD2K-LINCS Data Coordination and Integration Center, ISMMS, New York, NY, 10029; 7 Mount Sinai Center for Bioinformatics, ISMMS, New York, NY, 10029; 8 Healthcare Policy and Research, Weill Cornell Medical College, Cornell University, New York, NY, 10065; 9 Harris Center for Precision Wellness, ISMMS, New York, NY 10065; 10 Institute for Medical Engineering and Science, Massachusetts Institute of Technology, Cambridge, MA, 02139

**Keywords:** Drug discovery, chemogenomics, tensor completion, gene expression, drug repurposing

## Abstract

Gene expression profiling of *in vitro* drug perturbations is useful for many biomedical discovery applications including drug repurposing and elucidation of drug mechanisms. However, limited data availability across cell types has hindered our capacity to leverage or explore the cell-specificity of these perturbations. While recent efforts have generated a large number of drug perturbation profiles across a variety of human cell types, many gaps remain in this combinatorial drug-cell space. Hence, we asked whether it is possible to fill these gaps by predicting cell-specific drug perturbation profiles using available expression data from related conditions--i.e. from other drugs and cell types. We developed a computational framework that first arranges existing profiles into a three-dimensional array (or tensor) indexed by drugs, genes, and cell types, and then uses either local (nearest-neighbors) or global (tensor completion) information to predict unmeasured profiles. We evaluate prediction accuracy using a variety of metrics, and find that the two methods have complementary performance, each superior in different regions in the drug-cell space. Predictions achieve correlations of 0.68 with true values, and maintain accurate differentially expressed genes (AUC 0.81). Finally, we demonstrate that the predicted profiles add value for making downstream associations with drug targets and therapeutic classes.

## 1. Introduction

Genome-wide expression profiling of *in vitro* drug perturbations has proven to be useful for many aspects of drug discovery and development^1^. Applications include elucidation of drug mechanisms^2^, lead identification^3^, and drug repurposing^4, 5^. Despite this success, the capacity to leverage cell-specific responses has been hindered by limited data availability across cell types^6, 7^. To address this limitation, the Library of Integrated Cellular Signatures (LINCS) program^8, 9^ has greatly expanded the publicly available data to nearly one million profiles characterizing thousands of drugs exposed to dozens of cell types. However, this combinatorial space of drugs and cell types is vast, and many gaps remain in this space (see white space in Figure 3B). These gaps present difficulties both for large-scale analysis as well as for making cell-matched comparisons, e.g. between two drugs or between drug and disease. Therefore, we asked whether it is possible to leverage existing expression profiles to predict the remaining unmeasured profiles.

**Fig. 1:**
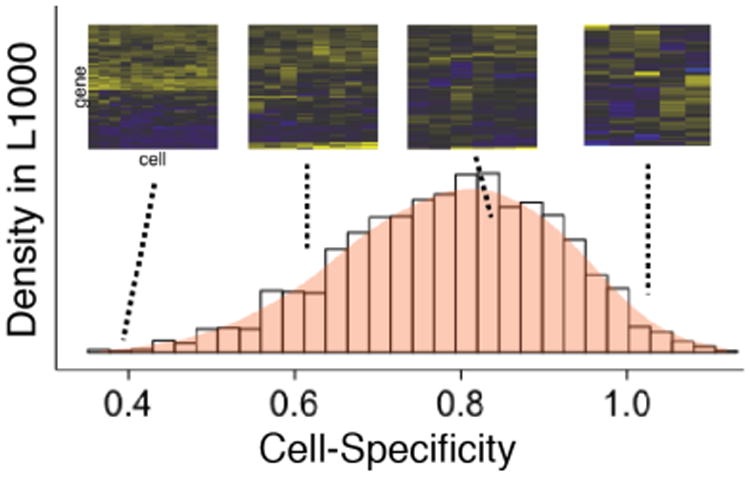
Distribution of cell-specificity of 2,130 drugs in the L1000 dataset. The cell-specificity is quantified per drug as the mean pairwise cosine distance between all of its cell-specific profiles, with a range of 0 (all cells identical) to 2 (perfect anti-correlation). Four examples are shown (L to R: homoharringtonine, terfenadine, dexamethasone, and JNJ-38877605). While some drugs induce very similar expression across cell types, the majority have higher cell-specificity corresponding to distinctive patterns in different cell types.

Expression responses to drug exposure are often highly cell-specific, e.g. due to differences in expression of drug targets. Indeed, we observe a high degree of cell-specificity for many drugs in the LINCS data (see Figure 1). The utility of such cell-specific gene expression has previously been demonstrated for a variety of applications. For example, a recent analysis^10^ found that LINCS expression profiles were more predictive of anti-cancer drug efficacy when using cell lines sharing a common lineage with the queried cancer type. Similarly, another study^11^ showed that using transcriptional similarity to predict drug-target interactions is more accurate when comparing drug profiles in the same cell line.

Prior studies have described methods to predict expression profiles using outside information. For example, Gamazon, et al. (12) predict tissue-specific expression profiles from genetic variants, but are limited to heritable variation in expression. Conversely, Lagunin, et al. (13) predict drug-induced expression responses from a drug's chemical structure, but are agnostic to cell type. There are also many techniques to impute missing entries of a gene expression matrix, generally using either local (e.g. nearest neighbors) or global (e.g. low-rank matrix approximation) information^14, 15^. However, most of these methods are not directly applicable to our setting, as they rely on having at least some measurements available in the target experimental setting.

Here, we draw inspiration from this prior work to solve a new problem: predicting *entire* expression profiles for cell-specific drug perturbations that have not yet been measured. Our two approaches are complementary in their use of local vs. global information. The local algorithm, *Drug Neighbor Profile Prediction* (DNPP) is inspired by K-nearest neighbors but adapted to this *de novo* prediction setting. The global algorithm, *Fast Low-Rank Tensor Completion* (FaLRTC)^16^ fills in the missing entries of a tensor using the observed entries. The underlying assumption here is that the data are low-rank, i.e. some small set of underlying factors (e.g. drug targets) explain most of the variation in the data.

We evaluate our methods along with two baselines using several approaches. We use cross-validation (CV) to measure correlation of true and predicted expression, as well as accuracy of differentially expressed genes (DEGs). We also study the dependence of accuracy on the amount of input data and explore the cell-specificity of our predictions. Finally, we demonstrate that the completed dataset adds value for downstream prediction of therapeutic classes and drug targets.

## 2. Methods

### 2.1. Notation and terminology

*T* refers to a tensor, with *T_d,g,c_* for drug *d*, gene *g*, and cell *c*. A colon subscript refers to all elements of that index. *C_d_* and *D_c_* respectively refer to the cell lines measured for drug *d*, and the drugs measured in cell *c*. Error bars in figures and text refer to ± one standard deviation. All correlations are Pearson's correlations, denoted by *r* or *cor*(·,·). ‘Drug’ refers to compounds represented in the data, including approved drugs, drug-like compounds, and tool compounds.

### 2.2. Data processing

**The LINCS drug expression data** (herein, the “L1000 data”) is measured on a targeted expression profilling platorm called L1000^17^. he platorm measures the expression of 978 “landmark” genes (roughly 1000, hence the name), selected to be maximally predictive of the other genes while being widely expressed across many cell and tissue types.

**Differential expression** computed from the level 3 L1000 data were downloaded from amp.pharm.mssm.edu/public/L1000CDS_download. The dataset was generated using the Characteristic Direction (CD) method^18^ and is validated and described more fully in^19^. Briefly, a CD was calculated for each replicate using linear discriminate analysis, to find the direction in gene space that best separates cases and controls. Replicates were averaged and normalized to unit length. Average cosine distance (ACD), i.e. the mean pairwise cosine distances between an experiment's CD replicates, was used to estimate significance. The null distribution of the ACDs was calculated per batch using random sampling (*n* = 10,000) of replicates in the same batch. A *p*-value for each profile (ACD *p*-value) was computed by comparing its ACD to the null.

#### Tensor construction

**Fig. 2:**
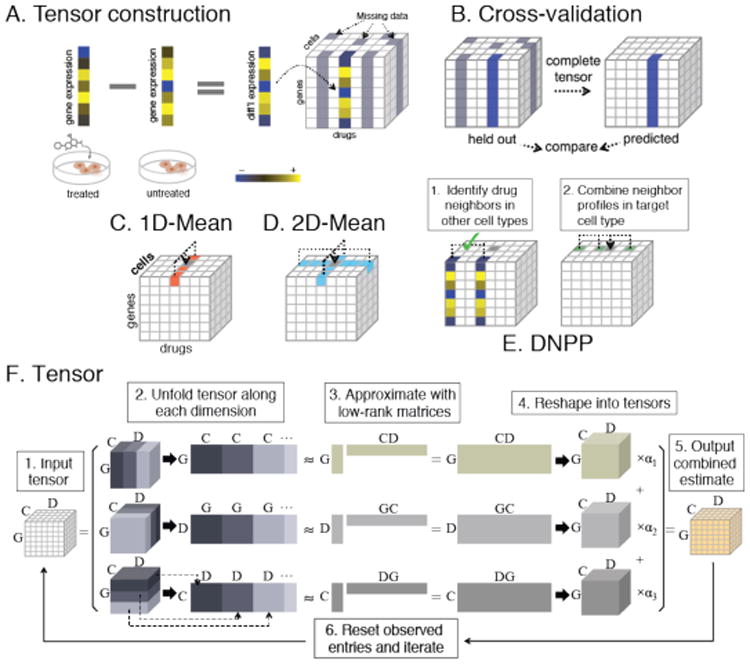
Schematic overview A. Expression profiles are compiled into a tensor of 978 genes × 2,130 drugs × 71 cell types. Profiles are either completely missing, in grey, or fully observed, denoted by both white and multicolor columns. B. CV setup, where entire profiles are held out. C-D Averaging baselines; target value is in grey and the averaged entries are colored. E. DNPP algorithm. Target value is in grey. Drug neighbors are identified by comparing profiles in other cell lines, then neighbor profiles in the target cell line are combined to form the prediction. F. FaLRTC algorithm. The data tensor is input on the left, and then unfolded in step 2 to form three matrices with dimensions G × CD (top), D × GC (middle), and C × DG (bottom). Each matrix is approximated using a spectral method and then reshaped into a tensor. The three tensors are then combined into one. Observed entries are reset to their initial values, and the process is iterated to minimize the matrix trace norms.

The 201,484 CD profiles (20,413 drugs, 72 cell types) were filtered to 34,716 profiles (6,928 drugs, 72 cell types) with ACD *p* ≤ 0.1 in order to remove the most unreliable data. Drugs and cell types with < 3 remaining experiments were removed, as well as duplicate drug id's corresponding to the same drug, for a final count of 25,672 profiles (2,130 drugs, 71 cells, 12.7% of all CDs). Profiles were averaged across all available concentration and time points, renormalized to have unit norm, and then arranged into a tensor (see Figure 2A). Of the 151,230 possible drug-cell pairs, the tensor contains 15,855, corresponding to 10.5% observation density. A smaller, more dense subset of this tensor was also used for some of the experiments, using the top 300 drugs and 15 cell lines, reaching an observation density of 71.4%. The tensor element *T_d,g,c_* is the *g*^th^ coordinate of the CD vector or drug *d* in cell *c*. All values lie in the range [-1,1] after normalization, where a positive [negative] value corresponds to up- [down-] regulation. The 10 cell lines with the most data are listed in Table 1, along with the corresponding tissue of origin and number of profiles (i.e. drugs) present. Most of the 71 cell lines are cancer cell lines, and represent a range of human tissues including skin, lung, brain, kidney, and prostate.

### 2.3. The Drug Neighbor Profile Prediction algorithm

The DNPP algorithm (Figure 2E) is an adaptation of K-nearest neighbors (KNN) to the *de novo* prediction setting. In other words, KNN normally requires at least some data present in the target condition in order to identify neighbors. To overcome this limitation, DNPP defines similarity between drugs^a^ instead of profiles. The similarity (*S*) between two drugs *d* and *d*′ is defined based on average correlation between the two drugs' profiles as measured in other cell types:

(1) $$S(d,d^{\prime })=\frac{1}{\left|C_{d}\cap C_{d^{\prime }}\right|}\sum_{c^{\prime }\in C_{d}\cap C_{d^{\prime }}}\text{cor}(T_{d,;,c^{\prime },}T_{d^{\prime },;,c^{\prime }}).$$

DNPP then estimates the profile for drug *d* and cell *c* as a weighted average of (up to) *K* profiles from cell type *c* corresponding to neighboring drugs. To generate a prediction for (*d*, *c*), drug neighbors of *d* are chosen only amongst drugs that have data in cell *c*, and hence neighbors can differ per cell type. Finally, the weights on the *K* profiles are chosen proportional to *S*(*d*, *d*′), normalized to sum to 1. We use *K* = 10 (CV results not shown).

### 2.4. The Fast, Low-Rank Tensor Completion algorithm

Since there are many tensor completion algorithms available, we benchmarked a variety of algorithms for speed and accuracy (see supplementary for details) and subsequently selected the FaLRTC algorithm. The FaLRTC algorithm^16^ is sometimes referred to herein as simply Tensor or ‘the tensor approach.’ We briefly describe the algorithm here, in a simplified form (see Figure 2F). Like most tensor completion algorithms, FaLRTC assumes that the data has some low-rank structure. While there is a notion of rank for a tensor^20^, this is in general hard to compute. Hence, FaLRTC resorts to low-rank matrix approximations instead. A three-dimensional tensor can be reshaped or ‘unfolded’ into matrices in three mathematically distinct ways^20^, i.e. a *D*×*C*×*G* tensor can be unfolded into a *D*×(*CG*), a *C*×(*DG*), and a *G*×(*DC*) matrix. The algorithm forms all three such matrices, and then performs low-rank matrix approximation via a spectral method. The prediction of missing values is based on a weighted combination of the three matrix-derived estimates, where these weights (*α_i_, i* = 1,2,3) are user-defined parameters, constrained to be positive and sum to one. Observed elements are reset to their true values, and then this process is iterated using gradient descent to minimize (an upper bound on) the matrix ranks. Due to the column-structured pattern of missing entries in our tensors, gene correlation structure is less useful for predictions than correlations in the other two dimensions, and hence estimates from the matrix (*G*×(*DC*)) that most strongly leverage gene correlations, were down-weighted by a factor of 100 relative to the other two (i.e. *α*_7_ ≡ *α*_1_/100 ≡ *α*_1_/100). This can be seen as an adaptation of the algorithm to the present setting defined by the column-structured pattern of missing entries.

### 2.5. Baseline averaging schemes

While many methods exist to impute randomly missing entries in a gene expression matrix, we are not aware of prior work predicting entire expression profiles without additional data inputs. Thus we use two simple baselines that make predictions by averaging relevant subsets of data. *1D-Mean* (Figure 2C) predicts missing expression profiles for each drug by averaging all profiles available for that drug in the tensor (i.e. across cell lines). *2D-Mean* (Figure 2D), combines the 1D-Mean average across cell lines with a similar average in the other dimension across drugs, i.e.

(2) $$2D\text{Mean}(d,g,c;\lambda )=\lambda \frac{1}{\left|D_{c}\right|}\sum_{d^{\prime }\in D_{c}}T_{d^{\prime },g,c}+(1-\lambda )\frac{1}{\left|C_{d}\right|}\sum_{c^{\prime }\in C_{d}}T_{d,g,c^{\prime }},$$

We use *λ* = ½ based on CV experiments (results not shown).

### 2.6. Cross-validation for predicting gene expression profiles

10-fold CV experiments were performed, where *entire expression profiles* were held out and then predicted (see Figure 2B), randomly selecting 10% of the profiles per fold. All of these predictions were compiled into a tensor, *T̂*, with the same dimensions and pattern of missing entries as the original tensor. Accuracy was measured as the Pearson correlation with truth (PCT). This is defined simply as PCT_Ω_ = *cor*(*T_Ω_*, *T̂*_Ω_), where Ω corresponds to some subset of the tensor, the correlation is taken element-wise, and missing entries are ignored. For example, Ω might correspond to an individual drug-cell profile, a CV fold, or the entire tensor.

### 2.7. Predicting drug targets and ATC codes

In order to build binary classifiers of drug-target interactions and Anatomic Therapeutic Chemical (ATC) classifications, drug profiles were compiled for all drugs represented in the data tensor, restricting to the top ten most-sampled cell lines (see Table 1). Measured profiles were used as is, and predicted profiles were generated using the DNPP method. The drug profiles and corresponding binary labels were used to train KNN, Random Forests (RF), and Regularized Logistic Regression (LR) models via the *caret* package^21^. For each experiment (i.e. one profile type, prediction task, model, and choice of either measured or completed dataset; see Figure 5A) a grid search was performed using 10-fold CV to select model hyperparameters (see supplementary). The cross-validated predicted probabilities from the selected set of parameters were recorded and then used to compute several versions of AUC scores. In the first set of experiments, AUCs are compared between (a) classifiers trained on the completed data, versus (b) the same classifiers trained on only the measured subset of profiles. Here, AUCs are calculated on the common set of labels corresponding to the measured drug profiles only, and results were excluded from the analysis when both AUCs were < 0.5. In the second set of experiments, AUCs were computed on two complementary sets of predictions from the same model trained on the completed data, where the complementary sets are the drugs with measured profiles, vs. the set of drugs for which only predicted profiles are available. Here, experiments were again excluded if both AUCs were < 0.5, or if either drug set (for the measured or predicted profile sets) had < 3 positive examples.

## 3. Results

### 3.1. Overall accuracy

**Fig. 3:**
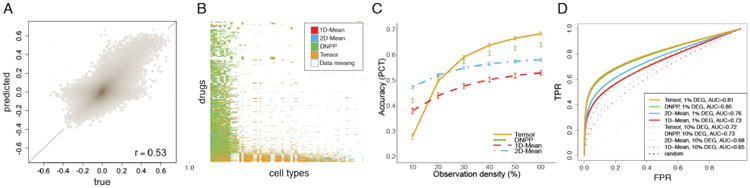
Prediction accuracy A. Scatterplot of Tensor-predicted vs. true values. B. Top-performing method per drug-cell profile in the tensor. C. Accuracy vs. observation density, where lower densities correspond to entire profiles being held out of the small tensor. D. ROC curves assessing prediction of DEGs. See text for details.

We start with an evaluation of the overall correlation between true and predicted values. Figure 3A shows a smoothed scatterplot of all Tensor (FaLRTC) predictions versus true values, where each point corresponds to a single, numeric entry in the tensor. The four methods achieved correlations (i.e. PCT, see Methods) of 0.53, 0.54, 0.46, and 0.40^b^.

### 3.2. Tradeoffs in accuracy across drug-cell space

While DNPP and Tensor have similar overall performance, we observe a clear tradeoff in accuracy between the two methods across different regions of the space. Figure 3B shows which method was most accurate (based on PCT) for each profile in the tensor. We see that for drugs with profiles in many cell lines (i.e. near the bottom), the tensor approach is usually the top performer, while in the region on the left where fewer cell lines but many drugs have been profiled, DNPP is generally superior.

### 3.3. Effects of varying observation density

Next, we studied the dependence of accuracy on the amount of input data by varying the percent of observed profiles in the small (and more dense) tensor. Observation density was varied by subsampling profiles in the tensor in 10% intervals from 10-60%, evaluating on a held-out set covering another 10% of the tensor. This sampling process was repeated 25 times generating the error bars in Figure 3C. At or above an observation density of 30%, Tensor had superior performance, while at lower densities, 2D-Mean was the top performer. We also observe that the tensor approach had a more dramatic improvement in performance with increasing density, reaching a mean PCT per fold of 0.68.

### 3.4. Accuracy of differentially expressed genes

We also evaluated the ability to predict DEGs in the unmeasured drug-cell experiments. To do this, we first identified DEGs in the measured profiles, and then thresholded expression values in the corresponding predicted profiles to generate ROC curves. More specifically, for each expression profile, a gene was considered a “true” DEG if its absolute expression value was at or above the *p*^th^ percentile relative to all genes in the profile, where *p* was set to either 1% or 10% (other approaches were also tried with little effect on the outcome; results not shown). ROC curves shown in Figure 3D were then generated by varying an analogous percentile threshold across the range 0-100% for the predicted profiles in the CV tensors, thereby defining a set of predicted DEGs for each profile at each possible threshold value. Each ROC curve represents aggregate results across all profiles in the tensor. The methods achieved area under the ROC curve (AUC) values of 0.81, 0.80, 0.76, and 0.73 at *p* = 1%. At *p* = 10%, a similar relationship between methods was observed (0.72, 0.73, 0.68, 0.65). For all four methods, AUC's were higher at the 1% threshold relative to the 10% threshold, and this pattern was observed more generally (results not shown), where smaller values of *p* correspond with higher accuracy. This is reasonable in that smaller percentile thresholds correspond to genes with stronger differential expression signals.

### 3.5. Analysis of cell-specificity

**Fig. 4:**
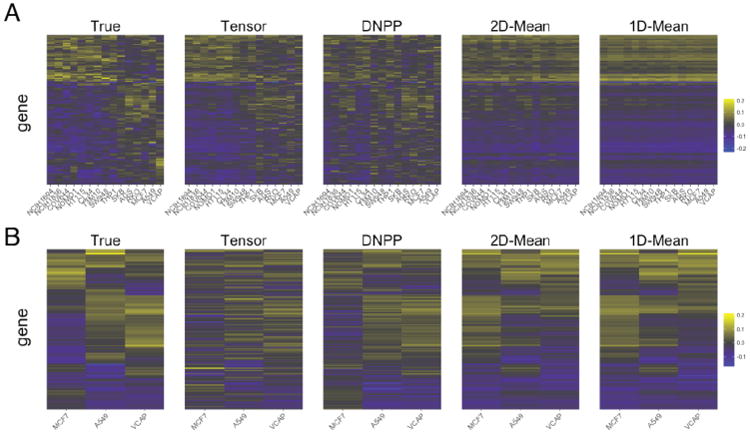
Cell-specificity of predictions A. True and predicted expression profiles for the compound M-3M3FBS (see text for details). Rows correspond to genes, and columns to cells. B. Analogous plots for Carbetocin, in the three available cell lines, MCF7, A549, and VCAP. See text for details.

While some L1000 drugs show very similar responses across cell types, others induce highly cell-specific responses. One such example is M-3M3FBS (herein “M3”), a PLC agonist that induces a variety of effects ranging from modulation of neutrophil function to apoptosis. The tensor contains M3 profiles in 15 different cell lines, shown on the left-most panel of Figure 4A. Responses cluster into two primary groups, with one group (on the left) enriched for down-regulation of both spindle pole genes as well as valine, leucine, and isoleucine degradation, perhaps indicating a pre-apoptotic response. The mean profile of the second group (A549, AGS, RKO, and MCF7 cells) is enriched for very different types of processes including up-regulation of Akt signaling, insulin signaling, and salivary secretion, all of which have established connections to PLC^22, 23^. Figure 4A shows that the tensor approach was able to accurately recapitulate these two classes of responses. DNPP, on the other hand, seems to “misclassify” some of the cell types into the wrong group, while 1D-Mean and 2D-Mean predictions are nearly identical across cell types.

Another example (Figure 4B) with highly cell-specific expression patterns is Carbetocin, an oxytocin analog. In contrast to the previous example, here DNPP outperforms the tensor approach. One explanation for DNPP's success with Carbetocin is that all three measured cell lines (MCF7, A549 and VCAP) are among the top five most-sampled cell lines in the tensor, and therefore have many drug neighbors from which to choose. On the other hand, M3 has data in many cell types, which is associated with better Tensor predictions. In addition to M3 and Carbetocin, two more examples are presented in the supplementary information, one (ABT-751) in which both methods do similarly well, and a second (GNF-2), where both have similarly poor performance.

### 3.6. Utility of completed data for downstream prediction of drug properties

**Fig. 5:**
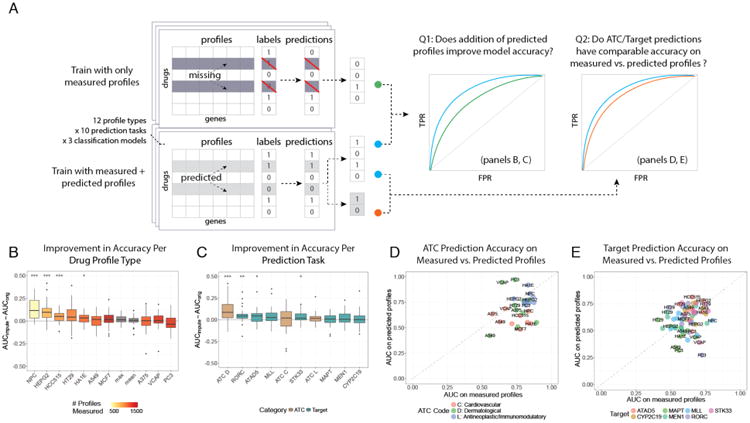
Utility of completed data for downstream predictions A. Illustration of experimental setup. Binary classification models are trained to predict drug targets and ATC codes, using either the measured subset of profiles (e.g. for a particular cell type) or the completed data, and cross-validated prediction scores are recorded. Then two types of ROC curve comparisons are made, as described in the text. B. Improvements in AUC per drug profile type, across experiments for different prediction tasks and models. C. Improvements in AUC per prediction task, across different profile types and models. D. ATC prediction accuracy on measured vs. predicted profiles, for different profile types. E. Target prediction accuracy on measured vs. predicted profiles. For both D and E, median values across models were computed to simplify the plots, but were kept distinct for all reported results.

In this final section, we aim to show that the completed data provides added value for downstream prediction of drug targets and therapeutic classes. To do this, we trained binary classifiers using the drug profiles as inputs, and designed experiments to address two questions (see Figure 5A). First, we asked whether classifiers trained on the completed data are of higher quality than those trained on only the measured subset of profiles. Second, we asked whether ATC and target predictions have comparable accuracy on measured vs. predicted profiles. Toward both of these aims, we identified the top 7 drug targets and 3 ATC classes (see Figure 5C) represented in the tensor, and trained classifiers for each of these tasks using 12 different versions of input drug profiles (cell-specific profiles from the top 10 most-represented cell lines in the tensor, as well as the mean and maximum value of each gene across these 10 cell lines). Finally, since our questions are focused on the value of the drug profiles and not about a specific algorithm, we included three different algorithms in our experiments (LR, KNN, and RF).

The results addressing the first question were generally positive. More specifically, of the 360 experiments (12 profile types × 3 models × 10 prediction tasks), after removing 21 experiments where no signal could be found, 223 (65.6%) showed an increase in AUC when training on the completed data compared with only the measured subset, with a mean improvement of 0.03 (*p* < 1e-8, paired *t*-test). Differences were also significant (*p* ≤ 0.01) for each of the models individually, with mean AUC improvements of 0.05 for LR and 0.02 for RF and KNN. The improvements also varied by profile type, as shown in Figure 5B. More specifically, we observed that cell types such as NPC (neural progenitor cells) that had fewer measured profiles available saw the most gains when including the additional profiles. Overall, four profile types (NPC, HEPG2, HCC515, and HA1E cell-specific profiles) showed significant AUC improvements across models and prediction tasks (adjusted *p* < 0.05, paired *t*-test), with two additional profile types (HT29 and *max*) reaching marginal significance, and none showing significant decreases. Figure 5C shows a similar analysis per prediction task. The median AUC difference was positive for all prediction tasks, reaching statistical significance for 4 out of 10: ATC *D* code (dermatological indications), and RORC, STK33, and ATAD5 targets, with MLL reaching marginal significance.

Figures 5D and E summarize the second set of experiments addressing the question of whether accuracy is comparable on predicted vs. measured profiles. While one might expect that accuracy would always be worse on the predicted profiles, this is not the case. We find instead that the results are mixed, and vary per feature and outcome. For example, predicting the ATC L code (antineoplastic and immunomodulating agents), had similarly high accuracy using either measured or predicted profiles, likely due to strong expression signals for this class of drugs, as well as high relevance of the cancer cell lines for observing antineoplastic effects. However, across experiments for the ATC codes, there was a mean loss of 0.08 AUC using the predicted profiles. On the other hand, in the case of target prediction, there was no significant loss of AUC across experiments. Interestingly, the predicted HT29 profiles had better accuracy than measured profiles for 19 of the 21 target prediction experiments (mean AUC improvement 0.12), perhaps indicating de-noising in the predicted profiles. Additionally, we found that for all 10 tasks, there were multiple profile types for which the AUC was higher on the predicted profiles.

## 4. Discussion

Expression profiles characterizing *in vitro* drug perturbations are useful for a variety of applications in drug discovery. While many thousands of such expression profiles have been measured, large gaps remain in the combinatorial space across drugs and cell types. Hence, we asked whether it is possible to leverage existing data from other drug-cell combinations to predict unmeasured profiles. We tested both local and global approaches, finding that predictions are not only accurate in an overall sense but preserve signal that is biologically and therapeutically relevant, e.g. maintaining accurate DEGs and signal to predict targets and therapeutic classes.

Both Tensor and DNPP almost uniformly outperformed the averaging baselines, with highly complementary performance between the two methods (Figure 3B). This complementarity is concordant with intuition in that, the global approach can leverage all information available and hence outperforms when a large amount of information is available per drug; whereas the local approach has better performance when many drugs neighbors are available. In addition to this complementarity, there are other tradeoffs. On the one hand, Tensor was able to “learn more” than DNPP with increasing observation density (Figure 3D). On the other hand, DNPP is conceptually simpler, uses only a single parameter, and requires less computation time.

In our experiments with ATC and target prediction, we note that the purpose is not to demonstrate state-of-the-art accuracy, but to show that the completed data adds predictive value to the LINCS L1000 drug profiles. Indeed, we observed many cases showing significantly improved accuracy, with no cases of significant decreases in accuracy. These results are likely explained by several factors. For cell-specific profiles, the completed data contains more profiles, and hence models can be trained with more labels. For the *max* and *mean* profiles, the incomplete data has heterogeneous cell-type availability per drug whereas the completed data is summarized across a uniform set of cell lines. Additionally, it is possible that the predicted profiles may, in some cases, have a stronger signal-to-noise ratio than their measured counterparts, which could explain, e.g. the high performance of the predicted HT29 profiles (Figure 5E) in multiple prediction tasks.

Our framework produces testable and usable predictions at the L1000 profile level. More specifically, each value corresponds to the differential expression (CD) value of one gene in one cell line perturbed by one drug. However, the CD values do not map directly to measurable gene-level quantities such as fold change. Therefore, we advise that, unless one compares predictions to the result of a CD analysis, predictions should either be treated at the level of a ranked list of genes, or thresholded to define DEGs.

We noticed while processing the L1000 data that roughly 2/3 of the > 20K drugs did not have any experiments with reliable (i.e. nominal ACD *p* < 0.1) measurements between replicates. While replicate consistency may improve with advances in data processing, it is likely that many of the drugs simply do not induce a strong enough expression response to overcome biological and technical sources of noise. We believe that this should be taken into consideration for any project working with L1000 drug profiles.

One limitation of this study is the lack of established baselines. The baselines used in this study were relatively basic, but help to demonstrate that our predictions outperform alternatives that might be considered safe and intuitive. While few methods currently exist for systematic prediction of cell-type specific drug expression profiles, we expect that the methods and results presented in this study would serve as useful baselines for future work on improved methods.

Several factors may have introduced bias into the results. First, almost all of the cell lines are cancer lineages, which may result in more similarity between cell lines than otherwise expected. Second, the selection of landmark genes may have biased the results. One line of thinking is that, due to the way these genes were selected, one would expect them to be relatively independent and therefore more difficult to predict than a random set of genes. If true, this would bias the results in a more conservative direction. Third, the presence of chemically similar drugs in the tensor could potentially make the prediction problem easier than otherwise. However, our analysis indicates that this bias is quite small (< 0.02 PCT difference), and we also verified that none of the drugs highlighted in Section 3.5 have structural cognates in the tensor (i.e. all Tanimoto coefficients are less than 0.5). Fourth, our CV experiments reduced observation density by 10%, and hence results would likely be further improved by using all available data. Finally, the L1000 data has highly imbalanced sampling across the drug-cell space (see Figure 3B), and this is likely a source of positive bias. Predictions made in the less-dense regions of the drug-cell space should therefore be used with caution and would likely benefit the most from methodological improvements.

There are many directions to explore in future work, grouped into a few categories. First, the data inputs could be expanded in a variety of ways. E.g., one could use the full, imputed transcriptome as opposed to only landmark genes. Also, more inclusive data filtering could be evaluated. The Broad Institute is also continuing to generate more data across this space; however, this will likely never be comprehensive, and hence we expect that this work will continue to be relevant. The second category of extensions are methodological, including: 1) nonlinear modeling; 2) use of auxiliary similarity information^24^; 3) addition of a time dimension to the tensor; 4) modeling measurement reliability; and 5) adopting a probabilistic framework. The final category of future work relates to applications. First, our approach could readily be applied and evaluated on many other biological datasets where data span at least three categorical axes. Such datasets include CMap^25^, with dimensions of drugs, genes, and cell types, and the Genotype-Tissue Expression (GTEx)^26^ and Braineac datasets^27^, each spanning individuals, genes, and tissues. Second, one could extend this framework to be able to prioritize the remaining experiments, e.g. using active learning, in order to optimally map out this transcriptional landscape across drugs and cellular contexts. Finally, another exciting direction would be to make possible ‘out-of-sample’ predictions^28^ which would be particularly useful when measurements are difficult to obtain (e.g. for human *in vivo* brain tissue expression) but where related measurements could be obtained from more accessible tissues (e.g., neuronal cell types from induced pluripotent stem cells). This would likely require an integrative approach leveraging additional datasets and metrics^24^ (e.g., cell line genetic similarity as auxiliary data for tensor completion).

To the best of our knowledge, this work is the first attempt at prediction of expression profiles using only expression from related experimental conditions. Hence, we consider this work to be a compelling proof-of-concept demonstrating the feasibility and value of such predictions. It is our hope that completing the space across drugs and cell types will enable new types of analyses and predictions of cell-specific drug action that could lead to translational insights and applications.

## Supplementary Material

2

## Figures and Tables

**Table 1:** The top ten cell types in the data tensor, along with tissue of origin and number of drug profiles available.

cell line	MCF7	VCAP	PC3	A375	A549	HA1E	HT29	HCC515	HEPG2	NPC
tissue	breast	prostate	prostate	skin	lung	kidney	colon	lung	liver	brain
# profiles	1505	1368	1340	1168	1139	1127	1022	934	798	441

## Footnotes

a We also tested a similar approach defining neighbors between cell lines, but the performance was not as strong.

b All results are reported in the following order: Tensor; DNPP; 2D-Mean; 1D-Mean.
