# Supplementary material for: Cell-specific prediction and application of drug-induced gene expression profiles
Source: Pac Symp Biocomput. Author manuscript; Available in PMC 2018 Jan 4. (PMC5753597)
Supplement: 2 [file NIHMS921438-supplement-2.pdf]

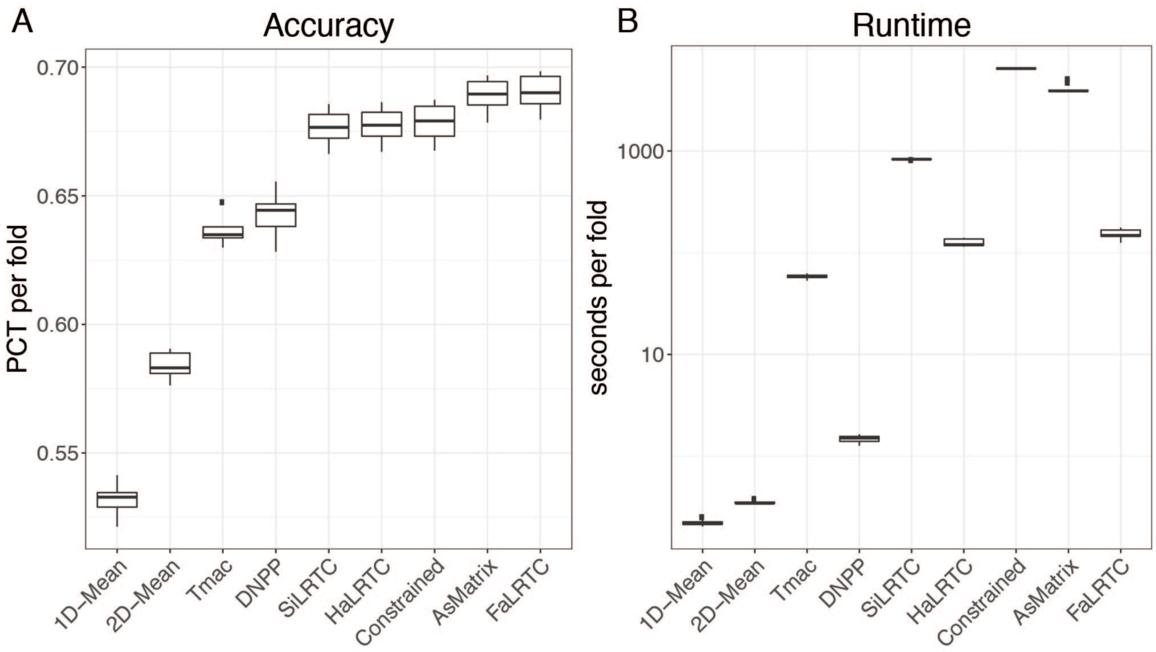

Figure S1: Six tensor completion algorithms were benchmarked for both speed and accuracy. The algorithms are: Tmac (Xu, et al., 2013); SiLRTC, HaLRTC, and FaLRTC (Liu, et al., 2013) and 'Constrained' and 'AsMatrix' (Tomioka, et al., 2010). MATLAB code for all algorithms was downloaded and used without any algorithmic changes. Some hyperparameters were hand-tuned while others were kept at default values (see Table S3). 10-fold CV experiments were run on the small tensor (300 drugs by 15 cell lines). Results and runtimes are shown here, along with 1D-Mean, 2D-Mean and DNPP. FaLRTC was selected for further study due to its superior accuracy and efficiency. A. Accuracy (PCT) per fold for each algorithm. B The corresponding runtimes (in seconds) per fold. Notice that the *y*-axis is on a log-scale, so that 'Constrained' and 'AsMatrix' are more than an order-of-magnitude slower than FaLRTC.

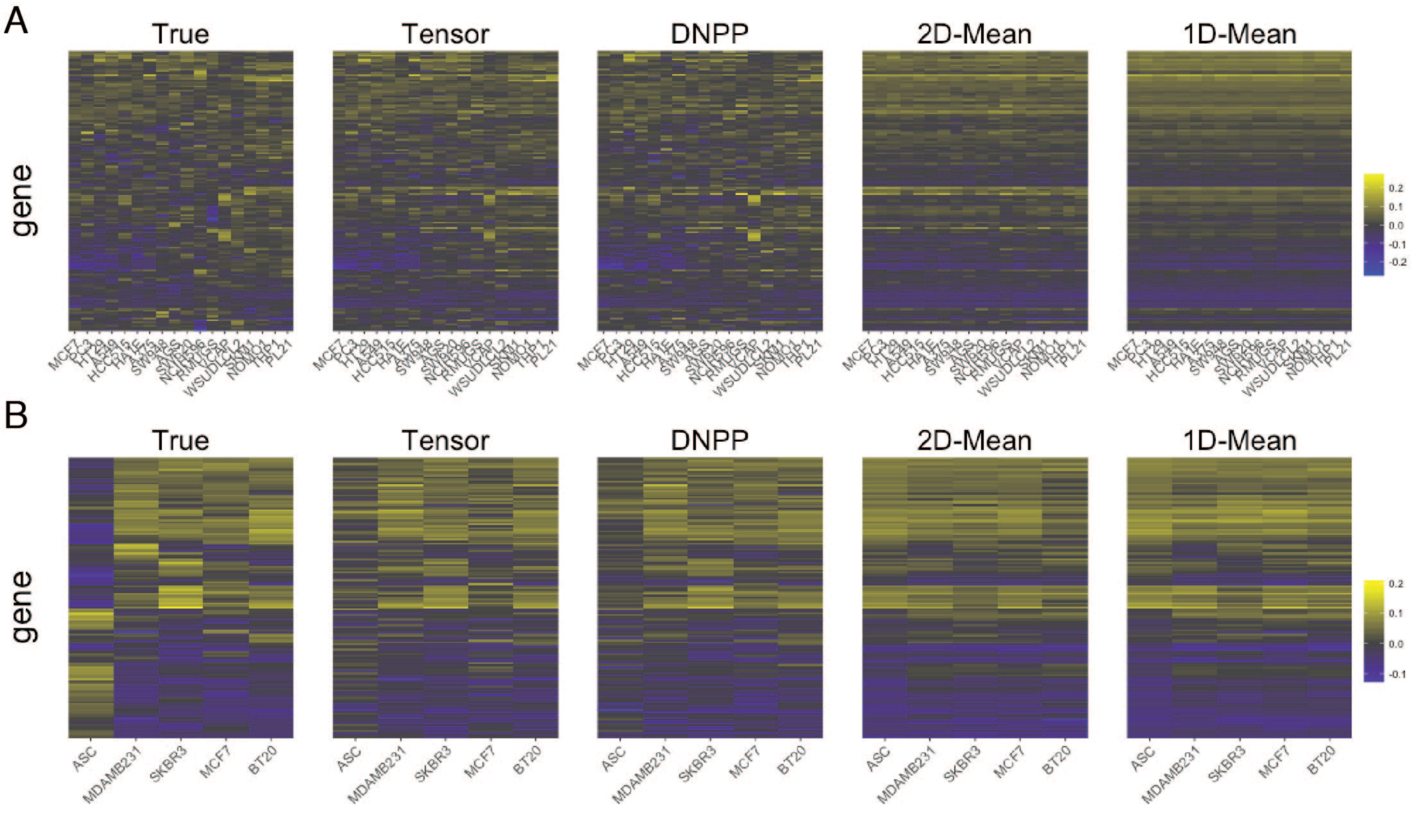

Figure S2: Two additional examples of cell-specific drugs where Tensor and DNPP have similar performance. A. ABT-751, a microtubule inhibitor. While not perfect, both Tensor and DNPP were able to recapitulate much of the detail in these highly cell-specific and complex expression responses, including patterns that are only observed in a single cell line. B. The cell-specific response to GNF-2, a Bcr-Abl inhibitor (Adrián, et al., 2006). In this case, 5 cell types have been measured, including ASC (fat) cells on the left, and four breast cancer cell lines. We observe a striking anti-correlation between the responses of the fat cells versus the breast cancer cells, with the latter response enriched for several cancer-related processes, consistent with recent connections between Abl kinases and solid tumor cancers (Greuber, et al., 2013). In this case, all methods did well in predicting the breast cancer responses, but none captured the anti-correlated profile of the fat cells. However, we do observe that both Tensor and DNPP predictions for ASC cells are qualitatively different from the breast cancer predictions.

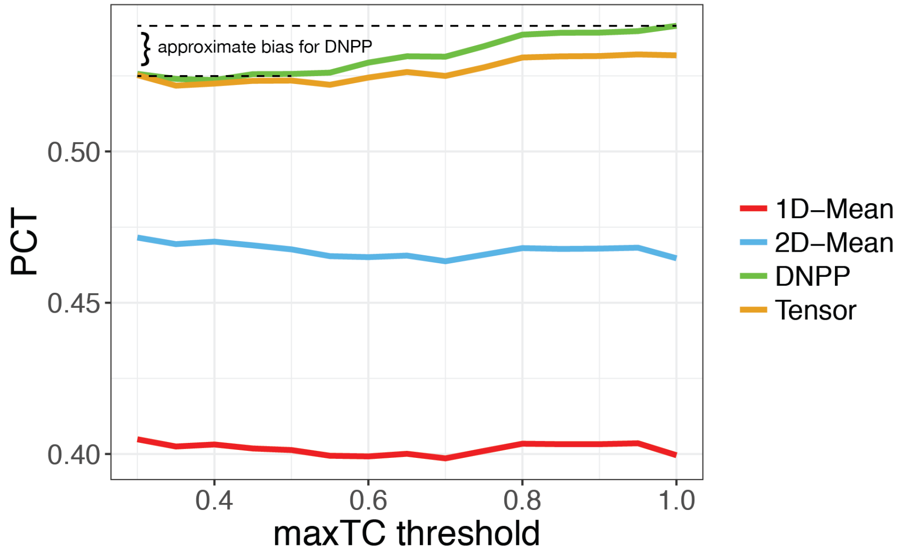

Figure S3: Analysis of bias resulting from the presence of chemically-similar drugs in the tensor. As mentioned in the discussion, chemically similar drugs can (but don't always) yield similar expression responses(Chen, et al., 2015), and this could lead to overly optimistic results, e.g. if a drug that is highly similar to the target drug is used in training. To quantify this bias, we first computed ECFP6 chemical fingerprints using the rcdk package. Then for each drug, we computed the maximum Tanimoto coefficient (maxTc) in relationship to any other drug in the tensor. Then, as shown in the figure, PCT accuracy was computed on the cross-validation results, either for the entire tensor as reported in the main manuscript (corresponding to a maxTC threshold of 1), or for the tensor restricted to drugs that have a maxTc less than some threshold. As expected, we see that 1D-Mean and 2D-Mean are not biased by chemical similarity, while DNPP and Tensor do reveal a slight bias, i.e. the results on the restricted subsets are a bit lower than for the entire tensor (dashed lines). The maximum bias revealed by this analysis is a PCT difference of 0.016 for DNPP (0.007 for Tensor), calculated by computing the difference between endpoints, as indicated in the figure.

Algorithm	Parameter	Value
FaLRTC	alpha	(1, 0.01,1) / 2.01
FaLRTC	mu	0.01
FaLRTC	C	0.5
FaLRTC	L_0	1 x 10^{-5}
FaLRTC	maxIter	20
FaLRTC	epsilon	0.1
HaLRTC	alpha	(1, 0.001, 1) / 2.001
HaLRTC	beta	1
HaLRTC	maxIter	500
HaLRTC	epsilon	0.05
SiLRTC	alpha	(1, 0.001, 1) / 2.001
SiLRTC	beta	(32, 32, 32)
SiLRTC	maxIter	500
SiLRTC	epsilon	1 x 10^{-10}
Tmac	estCoreNway	(10, 10, 3)
Tmac	maxIter	100
Tmac	tol	1 x 10^{-4}
Tmac	alpha-adj	0
Tmac	alpha	(1, 1, 1)
Tmac	rank-adj	(1, 1, 1)
Tmac	rank-max	(50, 30, 6)
Tmac	rank-inc	(1, 1, 1)
Tmac	rank-min	(1, 1, 1)
Asmatrix	alpha	(0.5, 0, 0.5)
Asmatrix	eta	32
Asmatrix	tol	5 x 10^{-2}
Constrained	lambda	0
Constrained	eta	32
Constrained	tol	5 x 10^{-2}
2D-Mean	lambda	0.5
DNPP	K	10

Supplementary Table 1: Hyperparameters used throughout the manuscript for all profile prediction methods.

Algorithm	Parameter	Value
regLogistic	cost	2^(-10:20)
regLogistic	loss	L2_dual
regLogistic	epsilon	0.1
parRF	mtry	10, 20, 30, 40
knn	k	1:10

Supplementary Table 2: Hyperparameters tested for each ATC/target prediction experiment (i.e. for each input type and output prediction task).

REFERENCES

Adrián, F.J., *et al.* Allosteric inhibitors of Bcr-abl–dependent cell proliferation. *Nature chemical biology* 2006;2(2):95-102.

Chen, B., *et al.* Relating Chemical Structure to Cellular Response: An Integrative Analysis of Gene Expression, Bioactivity, and Structural Data Across 11,000 Compounds. *CPT: pharmacometrics & systems pharmacology* 2015;4(10):576-584.

Greuber, E.K., *et al.* Role of ABL family kinases in cancer: from leukaemia to solid tumours. *Nature Reviews Cancer* 2013;13(8):559-571.

Liu, J., *et al.* Tensor completion for estimating missing values in visual data. *Pattern Analysis and Machine Intelligence, IEEE Transactions on* 2013;35(1):208-220.

Tomioka, R., Hayashi, K. and Kashima, H. Estimation of low-rank tensors via convex optimization. *arXiv preprint arXiv:1010.0789* 2010.

Xu, Y., *et al.* Parallel matrix factorization for low-rank tensor completion. *arXiv preprint arXiv:1312.1254* 2013.
